# Supplementary material for: Anatomic Investigation of Two Cases of Aberrant Right Subclavian Artery Syndrome, Including the Effects on External Vascular Dimensions
Source: Diagnostics (Basel). 2020 Aug 14;10(8):592. doi: 10.3390/diagnostics10080592 (PMC7459800; doi:10.3390/diagnostics10080592)
Supplement: Supplementary file 1 [file diagnostics-10-00592-s001.zip › Supplemental Figures Final Submission/Supplemental Materials Final Submission.docx]

Supplemental Figures

Mitchell H. Mirande, Madelyn R. Durhman, and Heather F. Smith

**
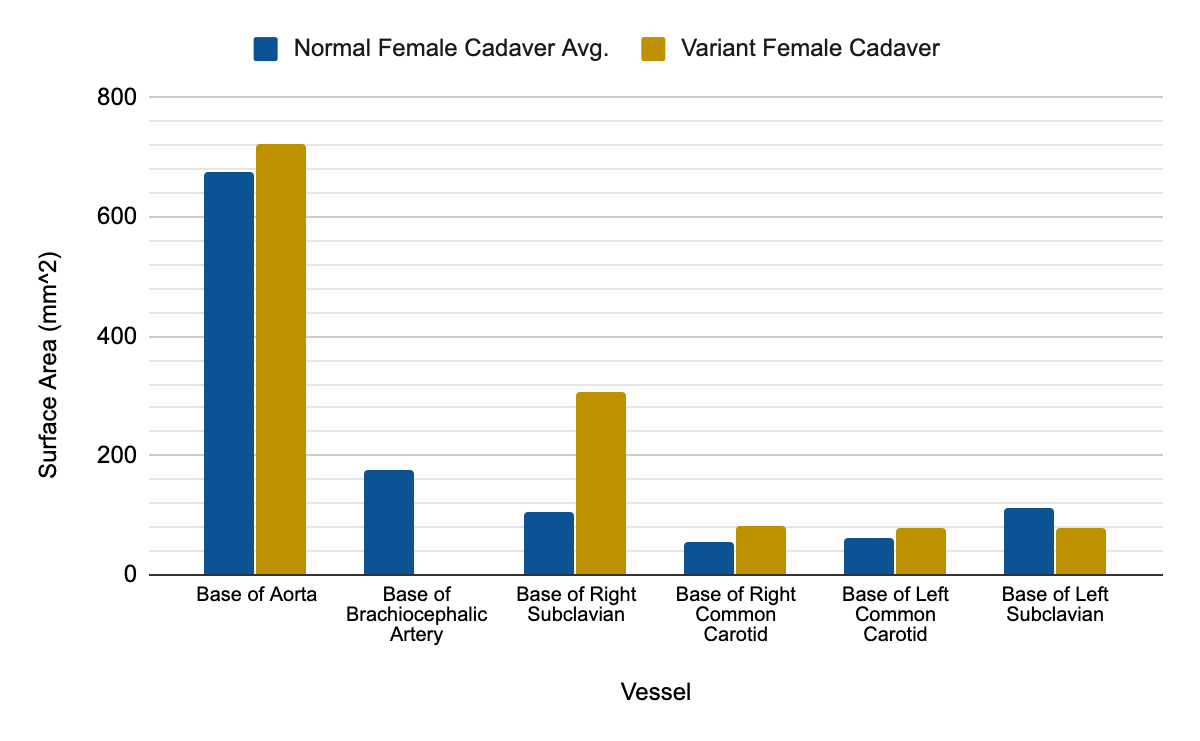
**

**Supplementary Figure 1.** Comparative results showing the surface area measurements of the normal female subjects vs the female ARSA variant subject. The surface areas at each location were averaged for each group.

**
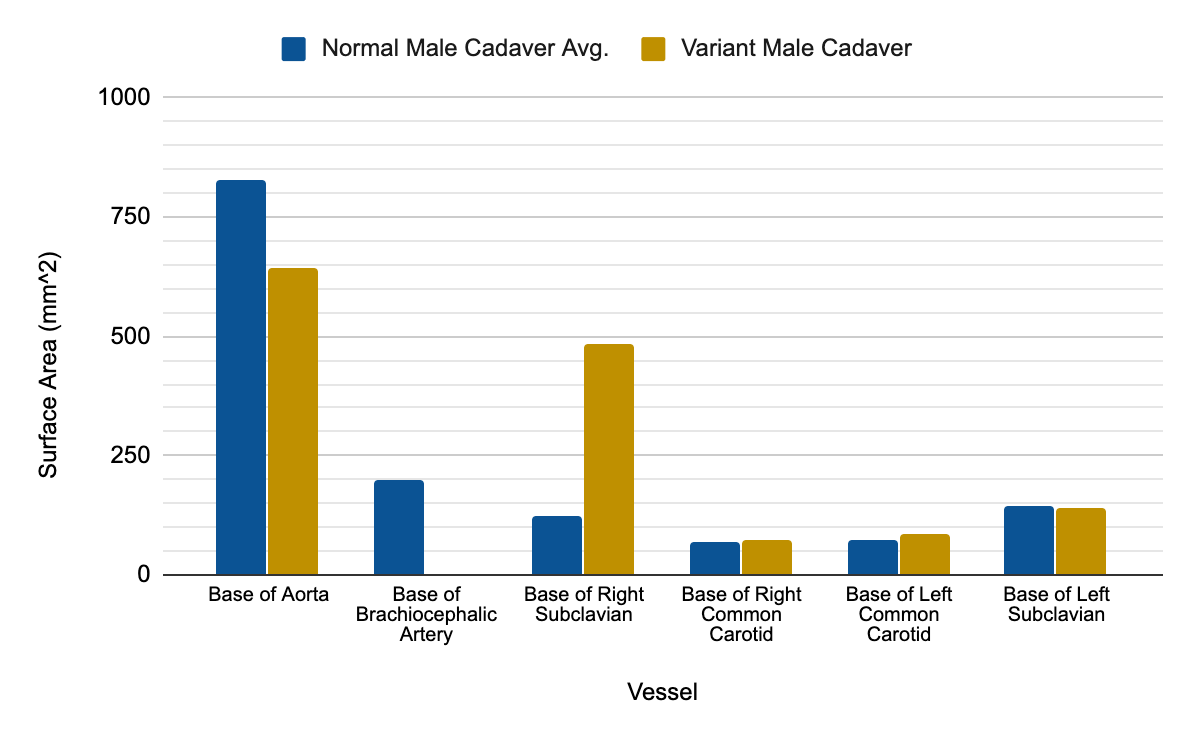
**

**Supplementary Figure 2.** Comparative results showing the surface area measurements of the normal male subjects vs the male ARSA variant subject. The surface areas at each location were averaged for each group.
